# Supplementary figures and images for: Introgression dynamics of sex-linked chromosomal inversions shape the Malawi cichlid radiation
Source: Science. Author manuscript; Available in PMC 2025 Jun 19. (PMC7617772; doi:10.1126/science.adr9961)

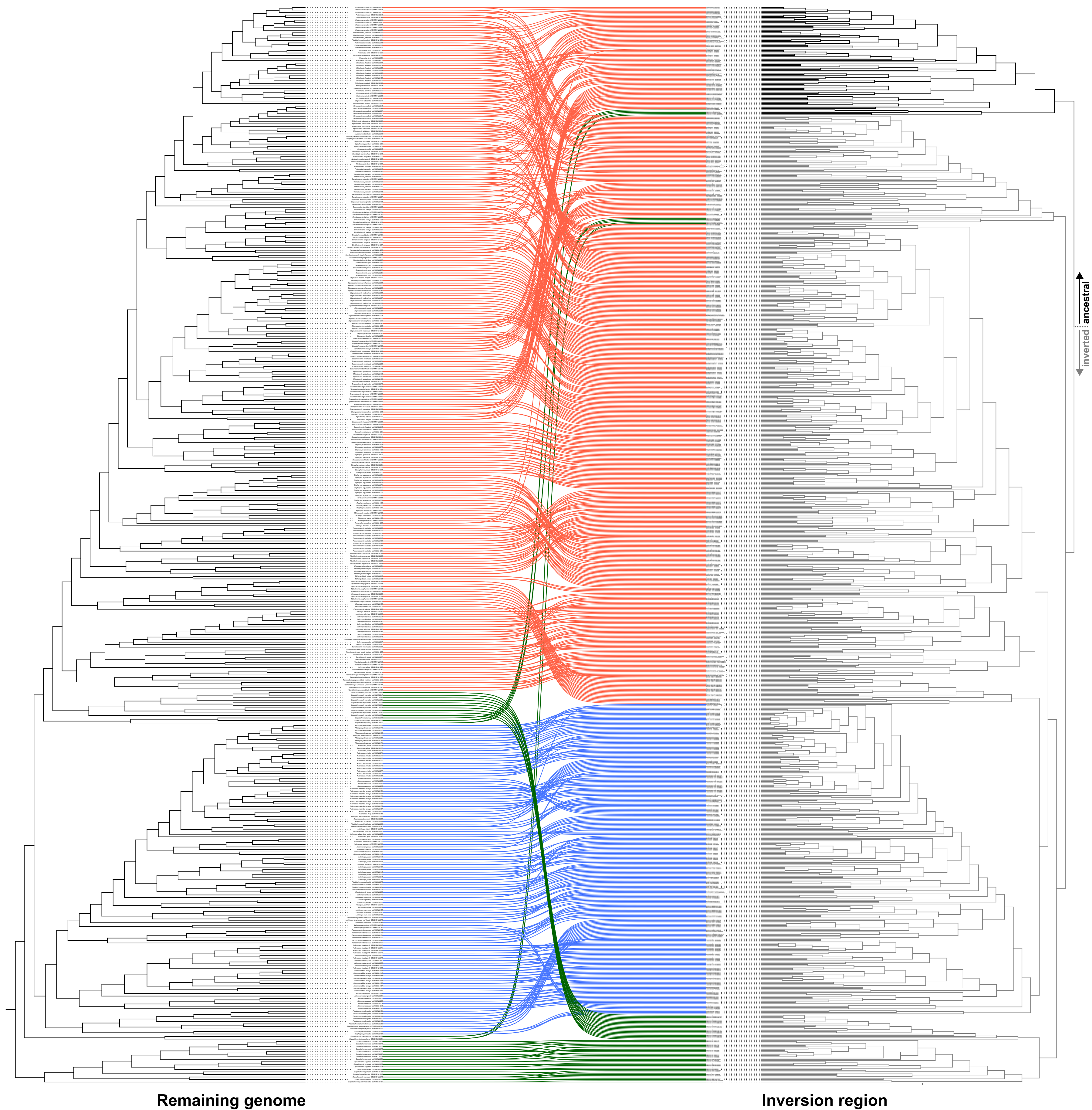

Supplement: Supplementary Data [file EMS206383-supplement-Supplementary_Data.zip › Data_S4.pdf]

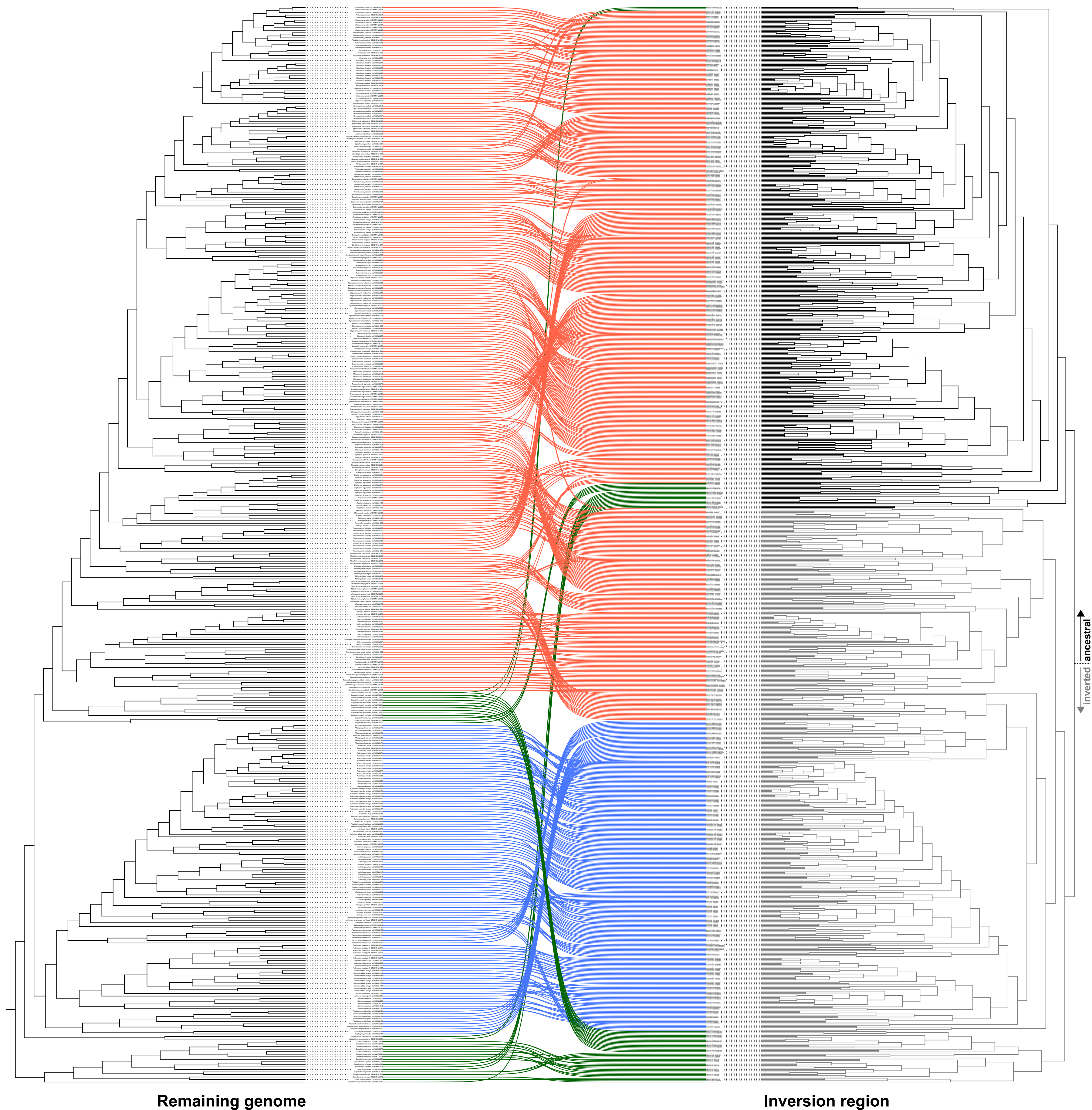

Supplement: Supplementary Data [file EMS206383-supplement-Supplementary_Data.zip › Data_S5.pdf]

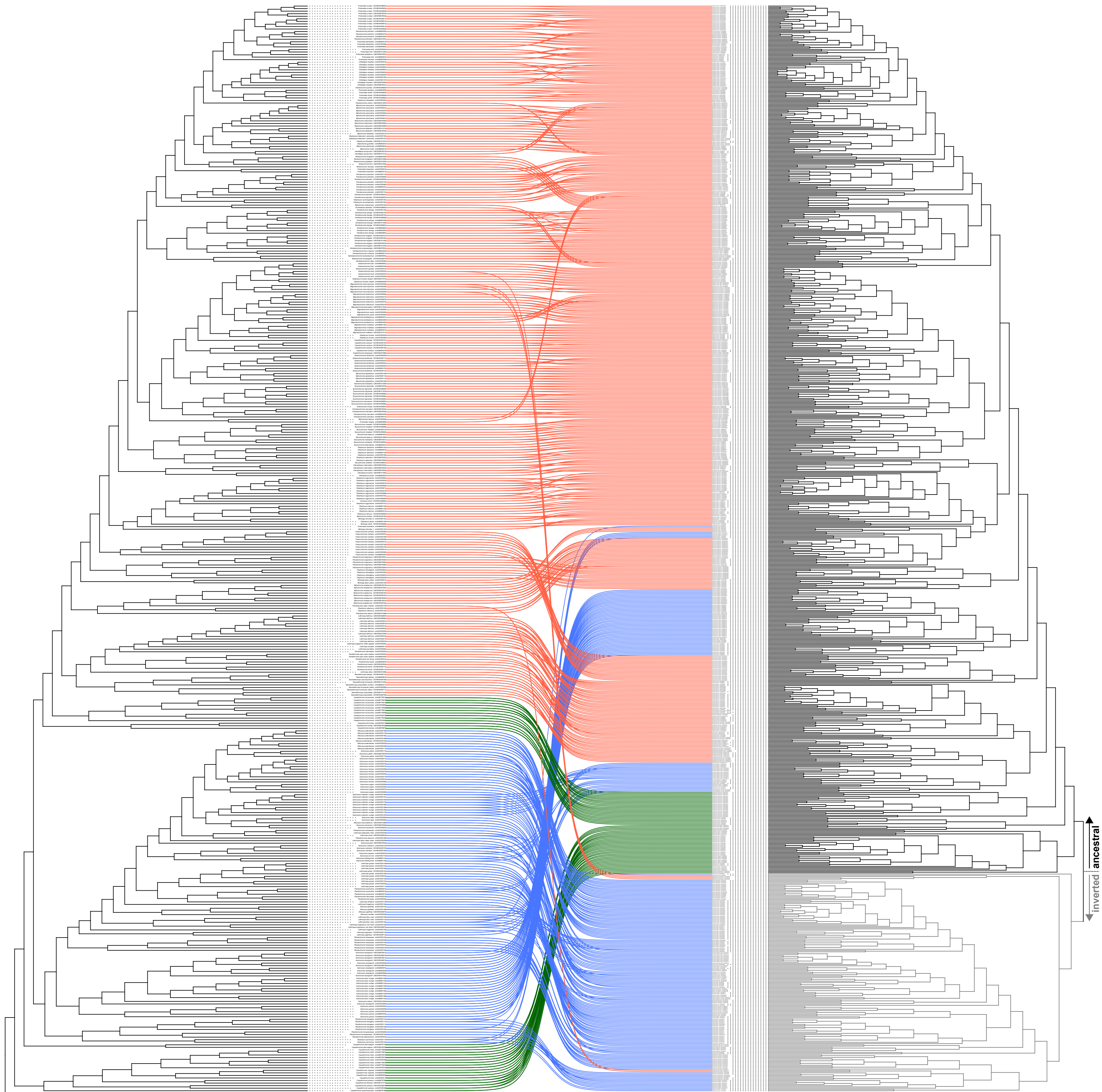

Remaining genome

Inversion region

Supplement: Supplementary Data [file EMS206383-supplement-Supplementary_Data.zip › Data_S6.pdf]

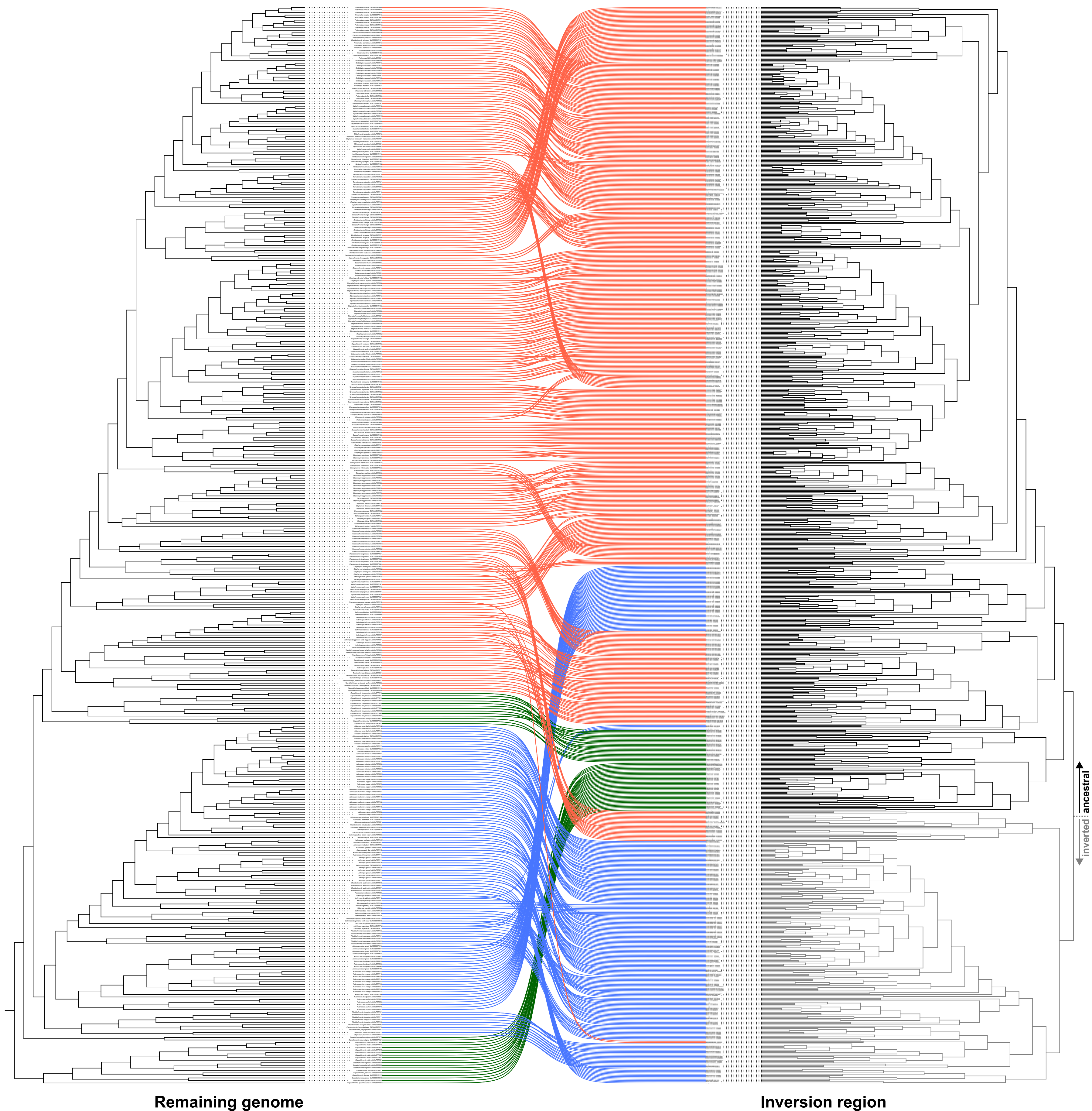

Supplement: Supplementary Data [file EMS206383-supplement-Supplementary_Data.zip › Data_S7.pdf]

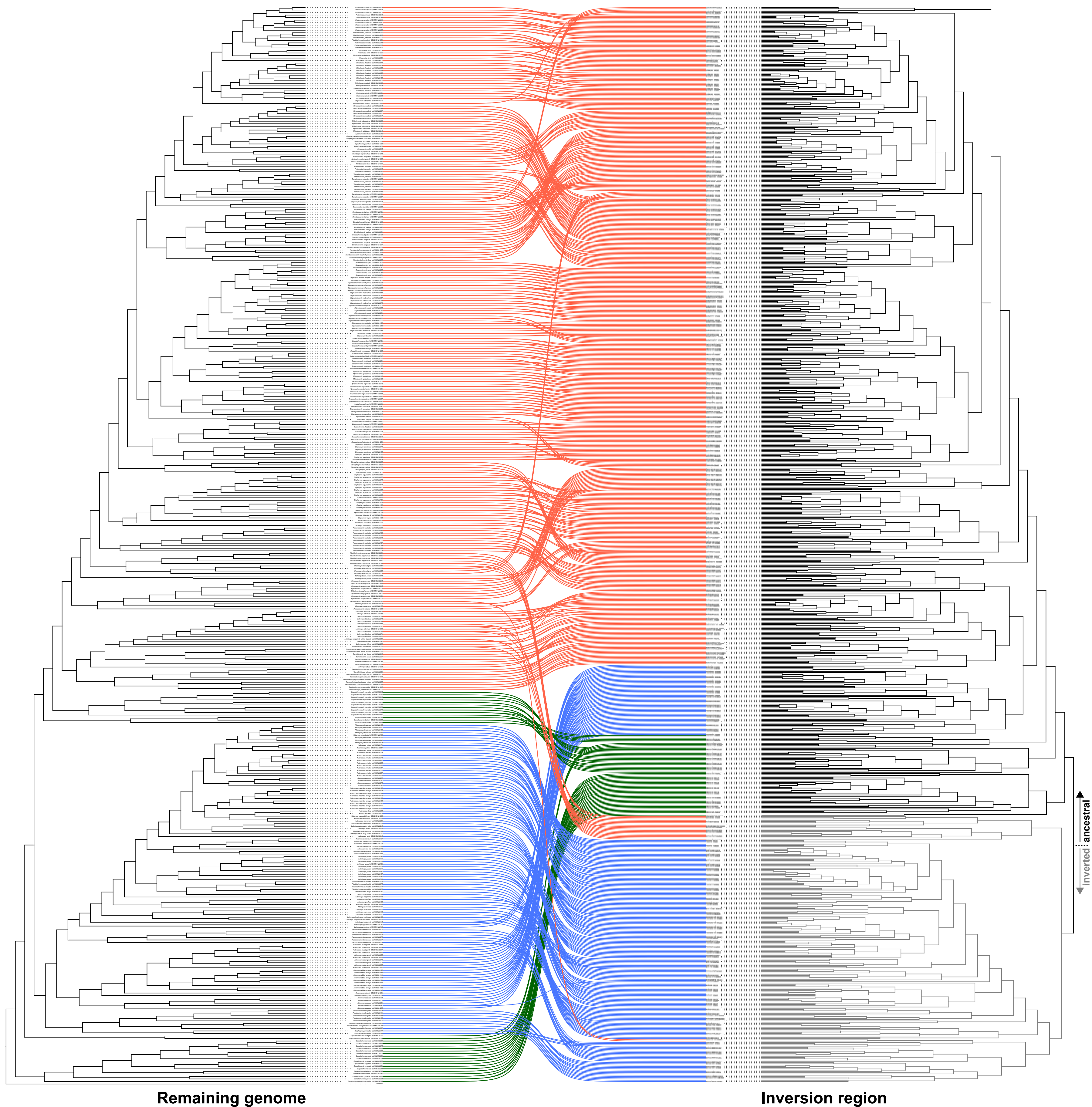

Supplement: Supplementary Data [file EMS206383-supplement-Supplementary_Data.zip › Data_S8.pdf]
